# Pulmonary blastomycosis presenting as primary lung cancer

**DOI:** 10.1186/s12879-018-3244-0

**Published:** 2018-07-18

**Authors:** Syed Mohammed Qasim Hussaini, Deng Madut, Betty C. Tong, Elizabeth N. Pavlisko, Wiley A. Schell, John R. Perfect, Nathan M. Thielman

**Affiliations:** 1 Duke University School of Medicine, Durham, North Carolina 27710 USA; 2 Duke-NUS Medical School, Singapore, 510568 Singapore; 3 Division of Infectious Diseases, Department of Medicine, Duke Global Health Institute, Duke University Medical Center, 310 Trent Drive, Durham, NC 27710 USA; 4 Division of Cardiovascular and Thoracic Surgery, Department of Surgery, Duke University Medical Center, Durham, NC 27710 USA; 5 Department of Medicine, Duke University Medical Center, Durham, NC 27710 USA

**Keywords:** Blastomycosis, Dimorphic fungus, Pulmonary mass, Posaconazole

## Abstract

**Background:**

Blastomycosis is an endemic mycosis in North America that is caused by the dimorphic fungus *Blastomyces dermatitidis*. The illness is a systemic disease with a wide variety of pulmonary and extra-pulmonary manifestations. The initial presentation of blastomycosis may easily be mistaken for other infectious or non-infectious etiologies.

**Case presentation:**

We present the case of a 52-year-old African-American male and former smoker that presented to his primary care provider with a 2-week history of non-productive cough, night sweats and weight loss. Initially diagnosed with primary lung malignancy, the patient was subsequently found to have pulmonary blastomycosis mimicking lung cancer. The patient underwent a successful course of treatment with posaconazole.

**Conclusions:**

Chronic blastomycosis can present with clinical and radiographic features indistinguishable from thoracic malignancies. There is no clinical syndrome specific for blastomycosis, thus a high degree of suspicion is required for early diagnosis. In this case report, we review recent evidence in radiographic features, diagnostic considerations and treatment of the disease.

## Background

Blastomycosis is a systemic pyogranulomatous infection that primarily presents as a lung infection, after inhalation of conidia of *B. dermatitidis*, with symptoms initially suggestive of an acute or chronic pneumonia. Hematogenous dissemination can frequently complicate the disease with extra-pulmonary manifestations that include the skin, bones, and the genitourinary system. Indeed, while the lungs serve as the primary portal of entry, the clinical manifestations of the disease can often be variable, with a delay in presentation that can last for weeks to months, between the appearance of an acute infection and development of chronic infection, during which a diagnosis can be missed or inappropriate treatments tried with little to no benefit. Blastomycosis also presents with varied radiographic features that can range from air-space consolidation, or mass-like lesions to diffuse military disease, further complicating the clinical picture. While culture and cytopathology remain the gold standards for diagnosing blastomycosis, recent emergence of tests that measure *B. dermatitidis* antigens have also proven useful especially in cases of unusual or delayed presentations. Once diagnosed, appropriate treatment of the disease with azole anti-fungals can result in rapid improvement in symptoms, and resolution of systemic manifestations and other radiographic features.

The current case report is a unique presentation of blastomycosis in North Carolina that initially began as a diagnostic challenge for the healthcare providers involved in the patient’s care. The overall aims of this case report include raising awareness of a common fungal disease that can present a diagnostic challenge; to review recent evidence including radiographic features, diagnostic considerations, and treatment of disease; and finally, to emphasize an appropriate diagnostic strategy and demonstrate a more cost-effective approach to management.

## Case presentation

A 54-year-old African American man initially presented to his primary care provider (PCP) with a two-week history of a non-productive cough and night sweats. The patient was a former smoker (~ 25 pack years) but otherwise had no other significant medical history and his HIV status was negative. Additionally, patient’s travel history as well as pet, home, and occupational exposures were non-contributory. The patient’s PCP prescribed a five-day course of azithromycin for acute bronchitis but he reported no improvement in symptoms. He was subsequently prescribed prednisone and albuterol for bronchospasms but the patient’s cough and night sweats worsened. In addition, the patient reported a 4.5 kg weight loss since the onset of his illness.

**Fig. 1:**
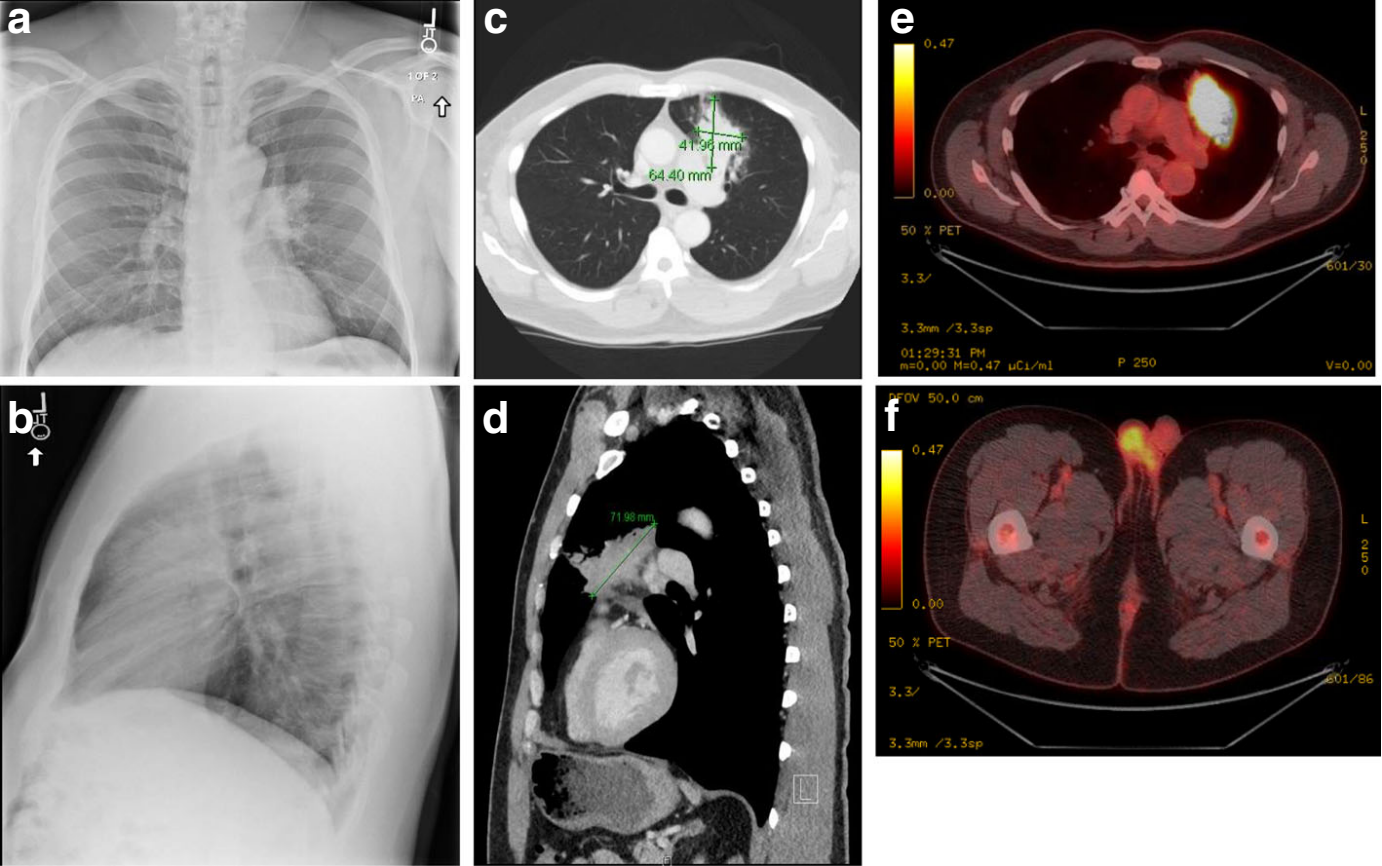
Representative images of suspected lung mass. **a-b** Left hilar mass with extension into the anterior segment of the left upper lobe as seen on PA (**a**) and lateral (**b**) plain films. **c-d** Left upper lobe abutting the pleural-pericardial reflection with preserved fat plane is seen on computed tomography (CT) scan. Numerous satellite nodules in the left upper lobe. **e-f** PET-CT images show FDG-avid left upper lung mass with multiple satellite nodules and ground glass opacities, with increased FDG uptake within multiple mediastinal left hilar lymph nodes (**e**). Focal uptake is Focal FDG uptake is also seen within a subcutaneous nodule along the superior left gluteal cleft/lower back (**f**)

Given his worsening symptoms, 3 weeks later he underwent a chest x-ray (CXR), which revealed a left hilar mass with extension into the anterior segment of the upper lobe (Fig. 1). A computer tomography (CT) scan revealed a 4.2 × 6.4 × 7.2 cm mass in the left upper lobe (LUL) with numerous satellite nodules concerning for primary lung malignancy. A positron emission tomography (PET)/CT revealed 18-fluoro-2-deoxyglucose (FDG) uptake in the LUL mass with a maximum standardized uptake value (SUV) of 24.3 as well as FDG uptake in left hilar lymph nodes with a maximum SUV of 4.3. There was also FDG uptake within a subcutaneous nodule along the superior left gluteal cleft with a maximum SUV of 21.1. The patient reported that this area was initially cystic looking and had been developing for a few weeks to months but was first noticed to be draining purulent fluid around the time of his illness. He was placed on doxycycline by his PCP after the PET/CT result for suspected pilonidal cyst. Bacterial culture from this lesion yielded no growth.

Given the concern for primary lung cancer, the patient was referred to cardiothoracic surgery to obtain a tissue diagnosis. His radiographic findings were thought to be consistent with primary lung cancer at a T2N2M0 clinical stage. The patient was taken to the operating room (OR), about 5 weeks after his initial presentation, and underwent a flexible fiberoptic bronchoscopy with biopsies of the LUL mass as well as cervical mediastinoscopy with biopsy of regional lymph nodes. These biopsies revealed no evidence of malignancy and thus the decision was made to pursue video-assisted thoracoscopy surgery (VATS) with lobectomy to obtain a definitive diagnosis and for therapeutic purposes. The patient underwent this procedure the following week. During the procedure, the mass was found to be adherent to the mediastinal pleura in the region of the phrenic nerve and thus not amenable to wedge resection. An intraoperative frozen section of the mass revealed necrotizing granulomatous inflammation. Given this information, the plan for resection was aborted and flexible bronchoscopy with washings for cultures was performed to rule out infection.

**Fig. 2:**
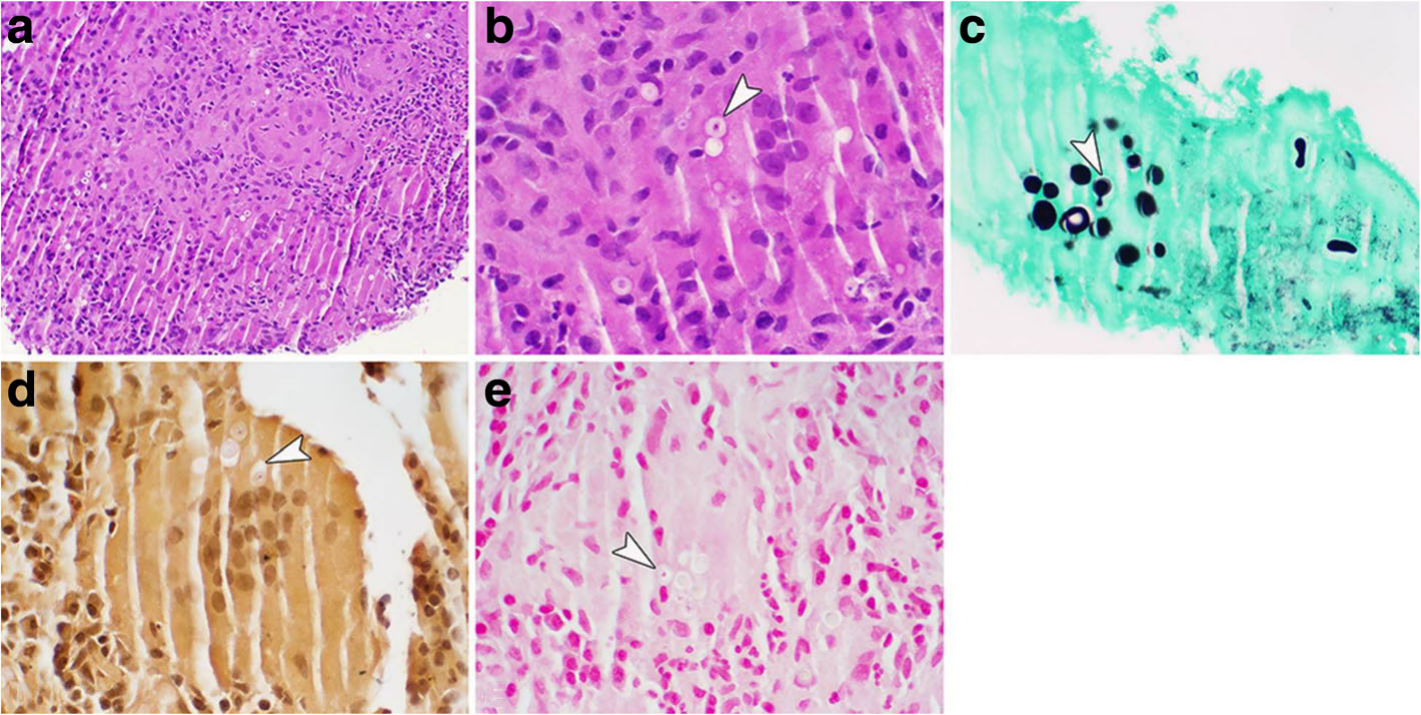
Photomicrographs from the left upper lobe mass following resection. **a** Non-necrotizing granulomatous inflammation with visible intracellular yeast. **b** High-magnification demonstrates thick, double contoured yeast cell wall with internal nuclear detail (arrow). **c** Grocott’s methenamine silver (GMS) stain highlights yeast up to 10 μm in which budding is evident and appears both narrow-based (arrow) or relatively broad (far right). **d-e** Mucicarmine (**d**) and Fontana-Masson (**e**) stains fail to demonstrate capsular mucin and melanin production (arrows), respectively. A-B H&E 200× and 600×, respectively; **c** GMS, 600X; **d** mucicarmine, 600×; **e** Fontana-Masson, 600×

The biopsy of the LUL mass again revealed no evidence of lung cancer on histopathology. However, yeast forms were seen on the biopsy concerning for *Cryptococcus*. Concurrently, the fungal cultures obtained from the patient’s bronchial washings had yeast on staining with growth of a mold on culture. These findings were consistent with a dimorphic fungus. This prompted mucicarmine and Fontana-Masson staining of the lung tissue which revealed 5 to 15 μm yeast forms with a double cell wall appearance consistent with *Blastomyces* (Fig. 2). A DNA probe was performed on the positive cultures and this confirmed that the fungus was *B*. *dermatitidis*. A fungal culture on his gluteal lesion also grew a mold consistent with *B*. *dermatitidis*. In addition, the patient had a positive B. *dermatitidis* urine antigen. Rest of the patient’s laboratory workup remained negative including Cryptococcus and HIV serology were negative.

**Fig. 3:**
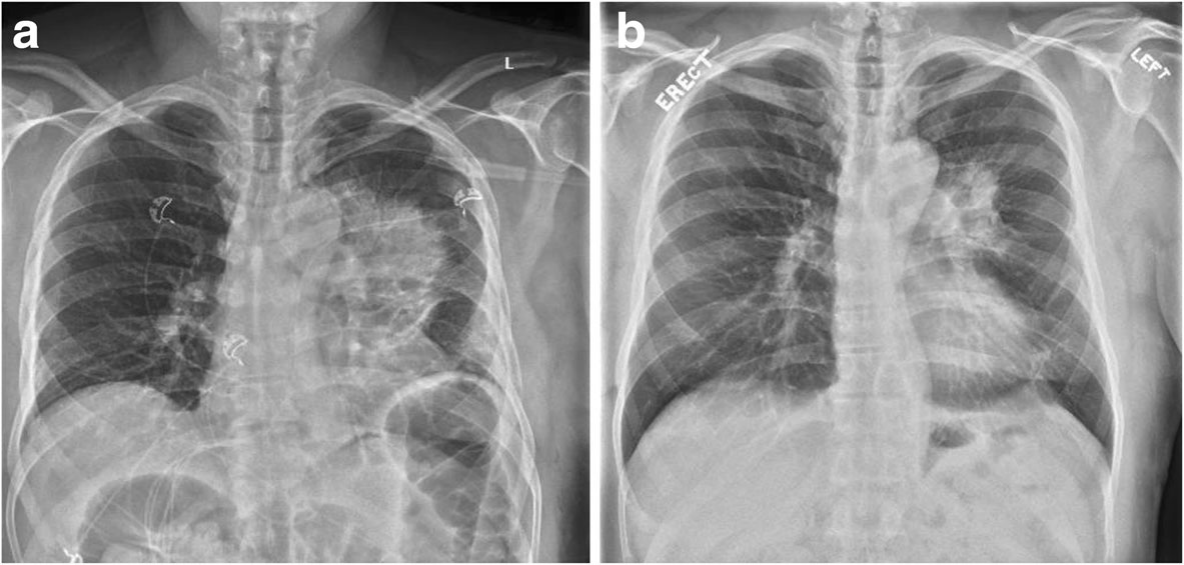
Chest imaging at discharge and following initiation of treatment. **a** Prominent left hilar mass is demonstrated in the left upper lobe of the lung at discharge. **b** There is a noticeable decrease in the size of the mass after 10 days with posaconazole treatment

**Fig. 4:**
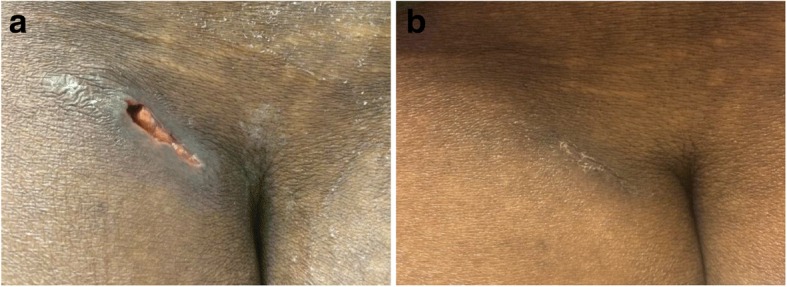
Imaging of left gluteal lesion after discharge and during treatment follow-up. Prominent lesion is visible at 1 week post-discharge (**a**) and has significantly healed at 6 weeks post-treatment initiation (**b**)

The patient was started on posaconazole for disseminated blastomycosis for a planned 6–12-month course. He started his course of posaconazole about 7 weeks after his initial visit to his PCP. A repeat CXR 1 month into posaconazole therapy revealed interval decrease in the size of the LUL mass (Fig. 3). At his two-month follow-up appointment, the patient reported resolution of his cough and night sweats. He lost about 10 kg over the course of his illness but reported that his weight was stabilizing while on therapy. On examination, his left gluteal cleft lesion had completely healed (Fig. 4).

## Discussion and conclusions

Blastomycosis is an endemic dimorphic fungus most commonly found in southeastern and south central states, especially those bordering the Mississippi and Ohio river basins [1, 2]. Recent reports have shown an increase in the incidence of blastomycosis in some regions with the highest incidence reported in Wisconsin. The yearly incidence rates of blastomycosis are 1 to 2 cases per 100,000. The initial causative mechanism of pulmonary infection is thought to be the inhalation of the conidia of *B. dermatitidis*. In nature, the organism exists in the mycelial form but converts to the yeast phase in the alveoli. The organism is typically found in soil that is warm and moist and rich in organic debris; thus, the major risk for infection is exposure to soil near waterways or in wooded areas. However, there is some evidence that some cases may arise in the home, especially in basements and attics [3]. While general preventive measures are difficult, individuals with weakened immune systems are at higher risk of blastomycosis.

Clinical manifestations of blastomycosis are quite variable and require a high degree of suspicion. The lungs serve as the portal of entry. Pulmonary infection can manifest as acute or chronic pneumonia, the latter being more common [1]. Acute pneumonia can present with fevers, fatigue, chills, and cough and may be difficult to differentiate from other infectious pneumonia [2]. Spontaneous resolution of acute infections have been reported although the frequency is unknown [4]. Diagnosis during the acute infection can be delayed for weeks to months, leading to the development of chronic pulmonary blastomycosis. During this period, patients typically receive multiple courses of antibiotics before a diagnosis is established. Symptoms during chronic pulmonary blastomycosis may include malaise, weight loss, night sweats, chills, fever, and possible hemoptysis^2^. These symptoms can be difficult to differentiate from other diseases. Indeed, a differential diagnosis during this time may include malignancy, tuberculosis, sarcoidosis, and other pulmonary infections or fungal diseases such as histoplasmosis.

Blastomycosis can present with a myriad of radiographic features including air-space consolidation, mass-like lesion, intermediate-sized nodules, interstitial disease, miliary disease, or cavitary lesions^5^. Air-space like consolidation is the most common radiographic finding and is often mistaken for a bacterial pneumonia. Masses on chest imaging are the second most common radiographic finding. They may be found in up to 31% of cases; typically in patients with a chronic presentation of the disease [5, 6]. These masses are often mistaken for lung malignancy, and in one case series from the Mayo Clinic, 55% of such masses were resected for suggestive bronchogenic carcinoma [6]. Similar to other fungal pneumonias, false positive PET-CT scan results can occur because of the recruitment of inflammatory cells, which have a high metabolic rate and thus increased uptake of FDG [7, 8]. In endemic areas, the differential for a lesion suspicious for lung cancer should include fungal pneumonias.

The most common site of extra-pulmonary manifestations of blastomycosis is the skin [1, 2]. Cutaneous lesions may be present in 40–80% of cases of blastomycosis and typically present as verrucous or ulcerative lesions [9–15]. Verrucous lesions have an irregular, raised border with possible exudate from an associated abscess in the subcutaneous tissue. These lesions can often be mistaken for squamous cell carcinoma. Ulcers have a more uniform and regular appearance^1^. Both lesions can be seen in the same patient.

In patients with pulmonary blastomycosis, there is a high positive yield with sputum cultures (86%) and an even higher yield with specimens obtained via bronchoscopy (92%) [16]. Although the diagnostic yield of wet preparations of sputum or pus is low, it should be considered given the low cost and simplicity. Serologic tests are available but have varying degrees of sensitivity and specificity and a significant amount of cross reactivity with other endemic mycoses [17]. Given these problems, serology is not thought to be the primary test for the diagnosis of blastomycosis. On histopathologic examination, the presence of pyogranulomas should prompt the possibility of blastomycosis. It may be difficult to visualize yeast forms with routine hematoxylin and eosin stain, thus specials stains are usually required.

Although culture and cytopathology remain the gold standards for diagnosing Blastomycosis, the detection of *B. dermatitidis* antigens may be a useful adjunct. The sensitivity of *B. dermatitidis* antigen assays for detecting antigenuria is between 85 to 93% and 57% for antigenemia [18–20]. The specificity is high, 99–100%, among healthy subjects or those without a known fungal infection. However, significant cross reactivity has been reported in patients with histoplasmosis as well as other fungal infection [19, 20]. Thus, a positive *B. dermatitidis* antigen assay should be interpreted with caution given the knowledge of known cross-reactivities. The utility of antigen detection assays for monitoring response to therapy remains an open question. A steady decline in detectable antigenuria and antigenemia has been shown in dogs treated with itraconazole for blastomycosis [21]. In humans, patients who responded to antifungal therapy have been shown to clear antigens, and the persistence of antigens may indicate treatment failure or non-adherance [22, 23]. A recent study has shown promising results with an antibody enzyme immunoassay using the *B. dermatitidis* surface protein Bad-1 [24].

Current treatment for blastomycosis includes azole antifungals such as itraconazole and flucanazole for mild to moderate infection with a lipid-based formulation of amphotericin reserved for severe life-threatening cases of the disease. With recent approval in the past decade of newer antifungals such as voriconazole and posaconazole, the number of available azole antifungals has expanded. Early studies of posaconazole compared it with amphotericin B, itraconazole, and flucanazole in mice infected with *B. dermatitidis*, and showed potent posaconazole activity with an MIC at which 90% of the isolates were inhibited of 0.06 mg/mL [25]. While larger trials are lacking, posaconazole has since been successfully used in treating patients with Blastomycosis [26]. We have had similar success at our center in treating blastomycosis with posaconazole. It is well tolerated, the oral tablet formulation is adequately absorbed, and the drug levels can be measured as well.

In conclusion, chronic blastomycosis, like other fungal infections, can occasionally present with clinical and radiographic features indistinguishable from thoracic malignancies. There is no clinical syndrome specific for blastomycosis, thus a high degree of suspicion is required for early diagnosis. Definitive diagnosis requires visualizing the organism by histopathologic examination or obtaining a positive culture. Given the vastly different approach to treatment between fungal infections and malignancy, fungal infections, particularly in endemic areas, should be considered in the patient with a suspicious lesion negative for malignancy or failure to respond to treatment for a typical lung infection.
